# Bakody’s Test Positive Thoracic Outlet Syndrome Caused by Anomalous Muscle: A Case Report

**DOI:** 10.7759/cureus.33646

**Published:** 2023-01-11

**Authors:** Ryota Kimura, Takashi Kobayashi, Naohisa Miyakoshi

**Affiliations:** 1 Department of Orthopedic Surgery, Akita University Graduate School of Medicine, Akita, JPN; 2 Department of Orthopedic Surgery, Akita Kousei Medical Center, Akita, JPN

**Keywords:** supraclavicular brachial plexus block, cervical spine anomalies, bakody’s test, anomalous muscle, thoracic outlet syndrome

## Abstract

Anomalous muscle causes thoracic outlet syndrome (TOS). A 40-year-old man presented with numbness of the left upper extremity, similar to cervical spondylotic radiculopathy. He presented with a positive Bakody’s test. However, magnetic resonance imaging showed no significant changes in the cervical spine and revealed an anomalous muscle adjacent to the left brachial plexus. We diagnosed the muscle as the cause of TOS and performed a resection, which resulted in symptomatic improvement.

## Introduction

Thoracic outlet syndrome (TOS) is caused by compression of brachial plexus elements or subclavian vessels in their passage from the cervical area toward the axilla and proximal arm [1]. Muscle anomalies may compress brachial plexus elements or subclavian vessels. Many of these are interscalene space anomalies and the subclavius posticus muscle [2-4]. Bakody’s test (shoulder abduction relief test) is a diagnostic test for cervical spondylosis. The test is performed by asking the seated patient to place their hand on the symptomatic side on top of their head. If the symptoms dissipate or resolve, the test is positive. The test has a specificity range of 80%-100% and a sensitivity range of 26%-50% [5]. TOS often shows a negative Bakody’s test [6]. We present a case of TOS caused by a muscle originating from the nuchal ligament and terminating in the clavicle, for which Bakody’s test was positive.

## Case presentation

We present the case of a 40-year-old man with a three-year history of dull posterior neck pain and numbness of the left upper extremity. His symptoms worsened when the left hand was lowered and relieved when shoulder abduction/forward elevation/external rotation was performed. He had no limitation in range of motion or muscle weakness. He was a carpenter and had difficulty working due to these symptoms. The patient was treated with nonsteroidal anti-inflammatory analgesics, with no response; instead, the symptoms increased in severity and became persistent during the previous two months. The patient had no history of illness or trauma.

The Jackson and Spurling tests were negative, but Bakody’s test was positive. Cervical spondylotic radiculopathy was suspected; however, there were no findings on radiography of the chest and cervical spine. Magnetic resonance imaging (MRI) of the cervical spine revealed no findings.

**Figure 1:**
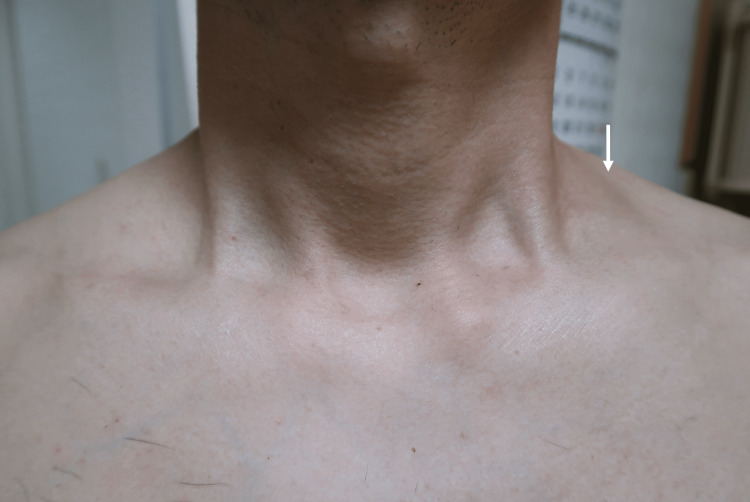
Body surface photographs of the neck. A mass is visible in the left supraclavicular fossa (white arrow).

**Figure 2:**
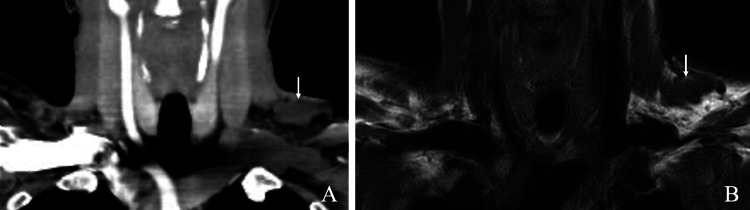
Plain computer tomography and magnetic resonance image. Plain computer tomography (A) and magnetic resonance image (B) of the neck show the anomalous muscle on the left brachial plexus (white arrow). It is not present on the right side.

**Figure 3:**
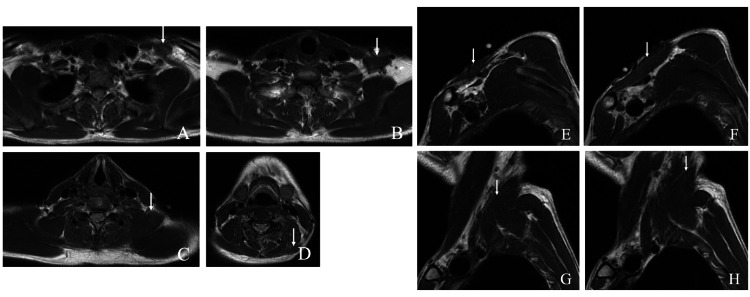
Magnetic resonance images of the neck T1-weighted magnetic resonance images of the neck (A, B, C, D, E, F, G, H) show the anomalous muscle. It originates from the nuchal ligament and stops in the middle one-third of the clavicular head side (white arrow).

On a repeated physical evaluation, a soft mass was revealed in the left supraclavicular fossa (Figure 1). The patient had rest pain in the left upper extremity in the drooping position, consistent with the same site, as well as tenderness and worsening numbness due to compression. There was no difference in the numbness and two-point discrimination test beyond the forearm between the radial and ulnar sides. No sensory impairment or muscle weakness was observed. There was no difference in the measured blood pressure in the upper limbs or palpated radial and brachial pulses. The Adson, Wright, Allen, and Roos test results were negative. A nerve conduction study revealed a velocity below 60 m/s in the brachial plexus trunk. Computed tomography (CT) with contrast of the chest revealed an anomalous muscle next to the left brachial plexus. MRI of the left brachial plexus showed the same pattern (Figure 2). It originated from the nuchal ligament and stopped in the middle one-third of the upper clavicular side (Figure 3).

**Figure 4:**
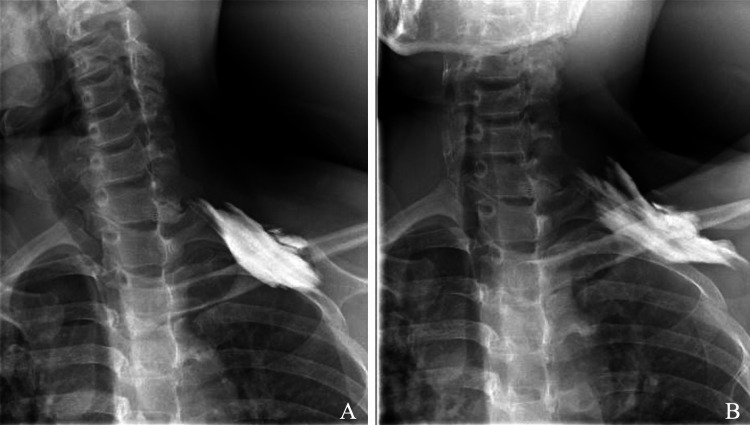
Radiographs after ultrasound-guided neurography of the brachial plexus. The contrast has moved caudally from the injection site, but the head side and head down positions show a blocked image (A). Upon left upper extremity elevation, which reduced symptoms, contrast migration to the head side is observed (B).

Ultrasound-guided neurography of the brachial plexus was performed, which was confirmed by fluoroscopy. The contrast agent moved caudally from the injection site, but the head side showed a blocked image, similar to the head-down position. When left upper extremity elevation was performed, symptoms were reduced and contrast migration to the head side was observed (Figure 4). Injection of a local anesthetic into the deep side of the muscle resulted in symptom relief. However, three hours later, the symptoms intensified.

Based on this course, TOS was diagnosed with an anomalous muscle as the compressive factor. We performed myotomy. Intraoperative findings included the identification of the brachial plexus under the muscle after myotomy. We instructed patients to restrict shoulder elevation for two weeks and heavy lifting for one month. The postoperative course was uneventful, and the patient was discharged on the third postoperative day. One week later, the patient presented no symptoms in the drooping position of the left upper extremity. He returned to work a month after surgery. The patient’s preoperative symptoms were completely relieved after three years.

## Discussion

The diagnosis of TOS is usually based on clinical symptoms and physical tests, such as the Adson, Wright, Allen, and Roos tests. This case was characterized by a positive Bakody’s sign and an anomalous muscle that had not been previously reported, which initially made us suspect cervical spondylotic radiculopathy.

**Figure 5:**
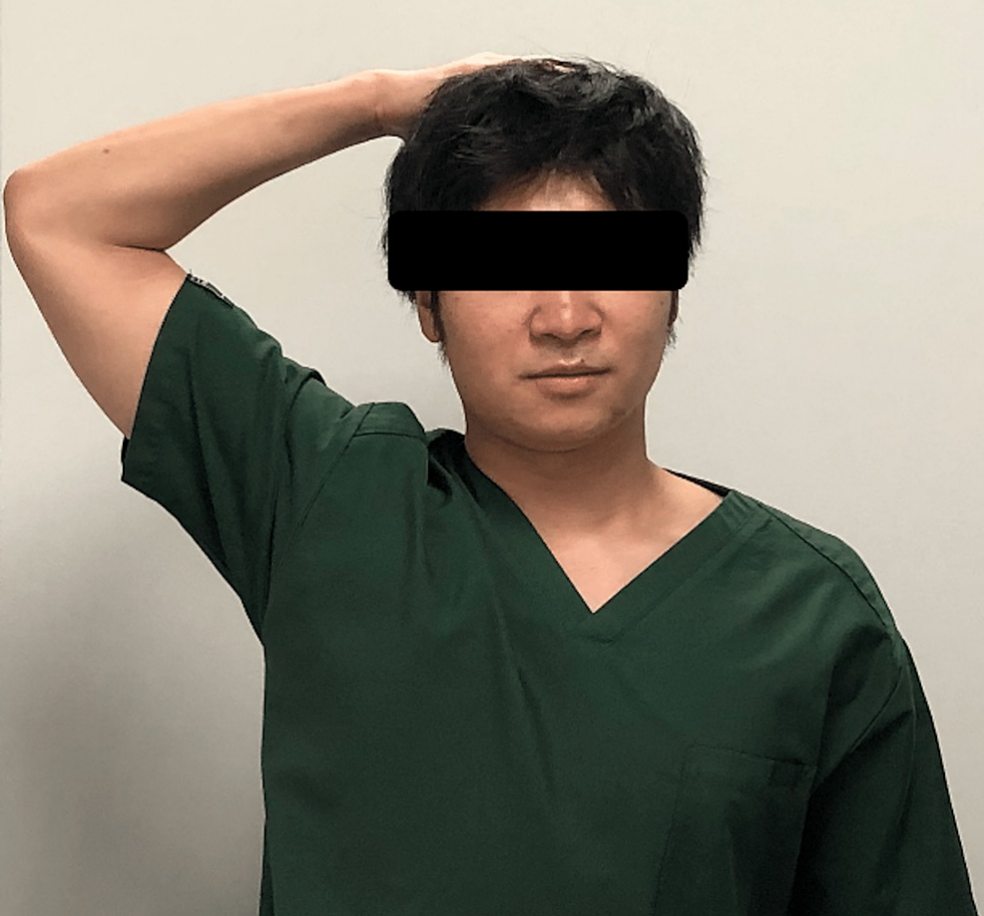
Bakody’s test (shoulder abduction relief test). The patient actively elevates the arm through abduction, so that the hand or forearm rests on top of the head.

In TOS, elevation of the upper extremity typically evokes symptoms because it leads to exacerbation of the stenosis. In the present case, the patient’s symptoms were relieved by elevation of the upper extremity. Considering the muscle trajectory, it is possible that elevation of the upper limb also elevated the clavicle, thereby reducing muscle tension and nerve compression. The patient is a carpenter and considers the possibility that muscle hypertrophy due to hard work may exacerbate the symptoms, even at middle age. Bakody’s test (shoulder abduction relief test) (Figure 5) was used to assess radicular symptoms, especially those involving the C4 or C5 nerve roots. Shoulder abduction/forward elevation/external rotation decreases the length of the neurological pathway and decreases pressure on the lower nerve roots. If the pain increases with the positioning of the arm, it implies that the pressure is increasing in the interscalene triangle.

Some congenital factors have been reported in thoracic outlet lesions [7]. When TOS symptoms occur, the possibility that the cause is an anomalous muscle should be considered. Visual examination and palpation were effective in the diagnosis of this muscle. Although 3D imaging using magnetic resonance neurography has been reported to be effective in diagnosing TOS [8], we could not use this technology. Therefore, we performed a dynamic evaluation using contrast media, which led to the diagnosis. Physical examination and imaging studies are important for the diagnosis of anomalous muscles. In such cases, brachial plexus decompression via myotomy is effective.

Levator claviculae are anomalous muscles that stop above the clavicle. It is estimated to occur in 2%-3% of cases, originating in the transverse process of the fourth cervical vertebra and stopping at the mid clavicle [9]. Levator claviculae have also been reported to contribute to TOS [10]. However, the origin of the muscle is different from that in the present case. No similar muscle cases have been reported in the past, and to the best of our knowledge, this is the first such report.

Though the TOS is more common in females [1], but since this case was a carpenter by occupation, he might had these symptoms because of his continuous posture of keeping the head in forward, drooping position. Exercise therapy is generally recommended for the TOS. Exercise therapy was proposed in this case as well, but the patient's work-related disability was too severe, leading to the choice of aggressive surgical treatment.

## Conclusions

If TOS is suspected, anomalous muscles should be considered as the cause of the symptoms. A positive Bakody’s test is mainly a sign of cervical spondylosis, but we should also suspect TOS. In TOS caused by anomalous muscles, resection of the anomalous muscles is an effective treatment.
